# Lipid emulsion attenuates vasodilation by decreasing intracellular calcium and nitric oxide in vascular endothelial cells

**DOI:** 10.1016/j.heliyon.2024.e37353

**Published:** 2024-09-03

**Authors:** Ling Chen, Hui Bai, Jing Zhao, Panpan Zhang, Xinhua Zhang, Dezhi Kong, Changzheng Dong, Wei Zhang

**Affiliations:** a Department of Pharmacology, Institution of Chinese Integrative Medicine, Hebei Medical University, 361 East Zhongshan Road, Shijiazhuang, Hebei Province, 050017, China; b Nursing Department, The Fourth Hospital of Hebei Medical University, China; c Department of Cardiac Ultrasound, The Second Hospital of Hebei Medical University, China; d Department of Neurosurgery, Hebei General Hospital, Shijiazhuang, Hebei Province, 050000, China

**Keywords:** Lipid emulsion, Calcium, Human endothelial cell, Vasodilation, Endoplasmic reticulum, Nitric oxide

## Abstract

Lipid emulsion (LE), a widely used parenteral nutrition, exhibits a well-documented ability to reverse the vasodilatory effects induced by acetylcholine in blood vessels. However, the specific mechanisms underlying this action are not yet fully understood. This study aimed to elucidate the mechanism by which LE reverses vasodilation in vitro through dose-response curve experiments, calcium imaging, and fluorescence assays. The results revealed a significant attenuation of acetylcholine (Ach)-induced vasodilation in rat thoracic aortic rings following LE exposure. In human aortic endothelial cells, pretreatment with LE significantly suppressed ATP-induced calcium elevation. This suppression persisted even after elimination of extracellular calcium with a calcium chelator. Moreover, LE pre-exposure reduced the intracellular calcium concentration ([Ca^2+^]_i_) elevation in endothelial cells following cyclopiazonic acid (CPA) treatment, suggesting enhanced endoplasmic reticulum (ER) calcium reuptake. Additionally, nitric oxide (NO) fluorescence assays showed a decrease in NO production upon ATP stimulation post-LE pretreatment of endothelial cells. Taken together, these results indicate that the reversal of vasodilation by LE may involve enhanced ER calcium uptake, leading to a reduction in intracellular calcium concentration and suppression of NO (key vasodilatory agent) synthesis.

## Graphical abstract

Schematic of the lipid emulsion (LE) effect on vascular response.

**Figure:**
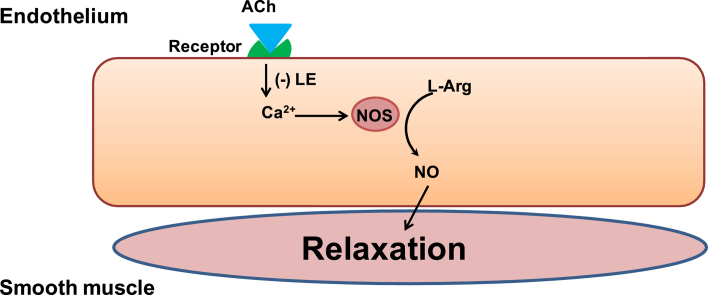

## 1 Introduction

Lipid emulsion (LE) therapy is a prevalent form of parenteral nutrition that supplies energy and essential amino acids to patients who are unable to receive oral nutrition. LE contains soybean oil, egg phospholipids, and glycerin [1]. Recent clinical and laboratory studies have found that intravenous LE can be used to treat systemic toxic effects, such as cardiovascular collapse caused by local anaesthetics, including bupivacaine, levobupivacaine, ropivacaine, mepivacaine, and lidocaine [2,3]. Lipid sink and fatty acid metabolism are two acceptable mechanisms for LE therapy [4,5]. The lipid sink theory suggests that a lipid compartment forms in the blood after LE infusion, which can absorb anaesthetics and reduce their free concentration in the blood [6]. The fatty acid metabolism theory states that increased fatty acids could be taken up by mitochondria, which could offset the inhibition of fatty acid metabolism caused by anaesthetics [7].

However, several recent studies have found that LE infusion may increase blood pressure and vascular resistance and inhibit local anaesthetic-induced vascular relaxation [[8], [9], [10], [11]]. Guo et al. discovered that pretreatment with LE alone or with noradrenaline reverses vasodilation induced by high doses of bupivacaine and ropivacaine in rat aortic rings [12]. Other studies have found that LE attenuates acetylcholine (ACh)-induced relaxation in isolated rat aortas [11,12]. In addition, the removal of endothelial cells abolishes all these effects of LE, indicating that the inhibitory effect depends on endothelial cells [12]. Endothelial cells, that form a continuous monolayer on the surface of the vascular lumen and play dual roles as essential biological barriers and active endocrine regulators. They synthesise and secrete various endothelium-derived vasodilatory factors such as nitric oxide (NO) to regulate vascular function [13].

To further elucidate the mechanism by which LE affects vascular function, we studied the effect of LE on vasodilation in isolated rat thoracic aortas and intracellular calcium levels in cultured human aortic endothelial cells (HAECs). Our results indicated that LE could reverse ACh-induced vascular relaxation by enhancing calcium reuptake into the endoplasmic reticulum (ER) and reducing NO production in endothelial cells.

## 2 Methods

### 2.1 Animals and ethic statement

Animal care and experimental procedures were performed in accordance with the National Institutes of Health Guide for the Care and Use of Laboratory Animals (2011). This study was reviewed and approved by the Ethics Committee of Hebei Medical University with the approval number: HebMU-20080026 Shijiazhuang, China.

Male adult Sprague–Dawley rats (8–9 weeks old; 250–300 g, N = 5), purchased from the Centre of Laboratory Animal Science at Hebei Medical University, were used for the study. Animals had free access to fresh water and food. All animals were housed under room temperature (22 ± 0.5 °C) and humidity (50 % ± 5 %) with 12:12 light dark cycle.

### 2.2 Chemicals

Phenylephrine hydrochloride (Phe, alpha-1 adrenergic agonist), ACh chloride, and cyclopiazonic acid (CPA, inhibitor of calcium-dependent ATPases) were obtained from Sigma-Aldrich Chemical Co., USA. Long-chain LE (20 %) was purchased from Sichuan Kelun Pharmaceutical Co. Ltd., China. It was diluted with Krebs-Henseleit (KH) solution to the required concentration. The reagents used to prepare the KH solution were purchased from Sigma-Aldrich (USA).

### 2.3 Thoracic aorta preparations

Rat thoracic aortic rings were prepared as described previously [12]. Male SD rats (N = 5) were euthanised using carbon dioxide. The thoracic aorta was dissected and placed into ice-cold Krebs-Henseleit (KH) solution containing 133 mM NaCl, 4.7 mM KCl, 1.35 mM NaH_2_PO_4_, 16.3 mM NaHCO_3_, 0.61 mM MgSO_4_, 7.8 mM glucose, and 2.52 mM CaCl_2_ (pH 7.4). To measure the aortic tension, the aortic rings were mounted on an isometric force transducer (MLT0380/D; AD Instruments Pty Ltd., Australia) connected to a data acquisition system (Powerlab/8SP; AD Instruments Pty Ltd, Australia) and maintained in a pre-warmed (37 °C) KH solution with continuous aeration of 95 % O_2_ and 5 % CO_2_. The basal tension of the aortic rings was set to 3.0 g. The aortic rings were equilibrated for 60-min prior to the administration of all test drugs.

### 2.4 Experimental protocols

The drug application procedures were performed as previously described [12]. Vasoconstriction of the aortic rings were assessed using the Phe cumulative concentration-response curve over a range of Phe from 0.0001 to 30 μmol/L. Vasodilation of the aortic rings was evaluated with Ach. After the maximum contraction of the aortic rings induced by Phe (0.0001–30 μmol/L) had reached a plateau, a series of Ach concentrations (0.0001–3.0 μmol/L) were added to the rings to generate a cumulative concentration-response curve. Endothelial function was considered normal if the maximum vasodilatory response to ACh was greater than 80 % of Phe vasocontraction and was used for subsequent LE experiments [12]. The LE-treated groups were pre-incubated with LE for 5 min before Phe and Ach treatment.

### 2.5 Cell culture

HAECs (ScienCell Research Laboratories Inc., USA, Cat. #6100) were grown on fibronectin-coated culture vessel (2 μg/cm^2^) in Dulbecco's modified Eagle's medium (DMEM) supplemented with 10 % fetal bovine serum (FBS), endothelial cell growth supplement (ECGs; ScienCell Research Laboratories Inc., USA, ECGS, Cat. #1052), 2 mmol/L L-glutamine, 100 IU/mL penicillin, and 10 μg/mL streptomycin. Cells were cultured in 35-mm dishes and grown in a humidified incubator with 5 % CO_2_. The HAECs were plated at a density of 1200 cells/cm^2^. Cells were used for the experiments between passage number 3 and passage number 10.

### 2.6 Calcium imaging

Calcium imaging was performed as previously described [14]. Cultured HAECs were loaded with fura-2-acetoxymethyl ester (2 μM; Invitrogen) in the dark for 20 min at 37 °C. After loading, endothelial cells were washed twice with 4- (2-hydroxyethyl)-1-piperazine ethanesulfonic acid (HEPES) buffer and placed in a recording chamber continuously perfused with HEPES buffer at room temperature [14]. The HEPES buffer contained 145 mM NaCl, 3 mM KCl, 2 mM MgCl_2_, 2 mM CaCl_2_, 10 mM glucose, and 10 mM HEPES (adjusted to pH 7.4 using NaOH). A Ca^2+^-free bath solution was modified by replacing the 2 mM CaCl_2_ with 4 mM MgCl_2_ and EGTA (0.1 mmol/L).

Calcium signals were generated at 340 and 380 nm using a monochromator (Polychrome V; TILL Photonics, NY). Calcium imagings were recorded at 1-s intervals using an EMCCD camera (Andor, Germany) mounted on a Leica DMI300B microscope. The calcium imaging ratio was calculated using a ratio metric imaging system (Metaflour, CA, USA). The ΔR (340/380)% was calculated using the following equation: ΔR = [P (340/380)-B (340/380)]/B (340/380)] × 100 %, as described previously [14].

### 2.7 Intracellular NO assay

HAECs were seeded in glass-bottom 96-well plates at 10,000 cells/well for 24 h prior to the assay. Cells were exposed to ATP, with or without 0.4 % LE, for 0.5–4 h in phenol red-free 5 % FBS medium. Cells were then washed with phenol red-free medium and exposed to 2 μmol/L DAF-FM DA (4-Amino-5-methylamino-2′,7′-difluorofluorescein diacetate, Thermo Fisher Scientific, Cat. D23844) for 40 min in the dark at 24 °C. The DAF-FM DA-containing medium was replaced with a fresh phenol red-free medium. The cells were then incubated in the dark for 10 min at 24 °C. Cells were visualised using a fluorescence plate reader (Microplate Readers; Biotek, Vermont, USA) to detect intracellular NO (excitation/emission 495/515 nm) [15].

### 2.8 Statistical analysis

Data are presented as mean ± SEM. Experimental sample size were determined based on prior studies [12,14,16,17]. Vascular tension alterations (g) were quantified as vasoconstrictive responses, while vasodilation triggered by ACh was expressed as the percentage of Phe-induced constriction. Statistical analyses were performed with GraphPad Prism 8.00 software (GraphPad Software Inc., USA). All data were first subjected to a normality test. The differences in the dose-dependent response curves with and without LE were evaluated using two-way analysis of variance (ANOVA). One-way analysis of variance (ANOVA) followed by Bonferroni's post hoc test was used to compare differences among three or more groups. The Student's *t*-test was used to analyze the difference between the mean responses of two groups. The type of statistical analysis for each experiment is indicated in the figure legend.

## 3 Results

### 3.1 Effect of different LE concentrations on Phe-induced vasoconstriction and ACh-induced vasodilation

**Fig. 1:**
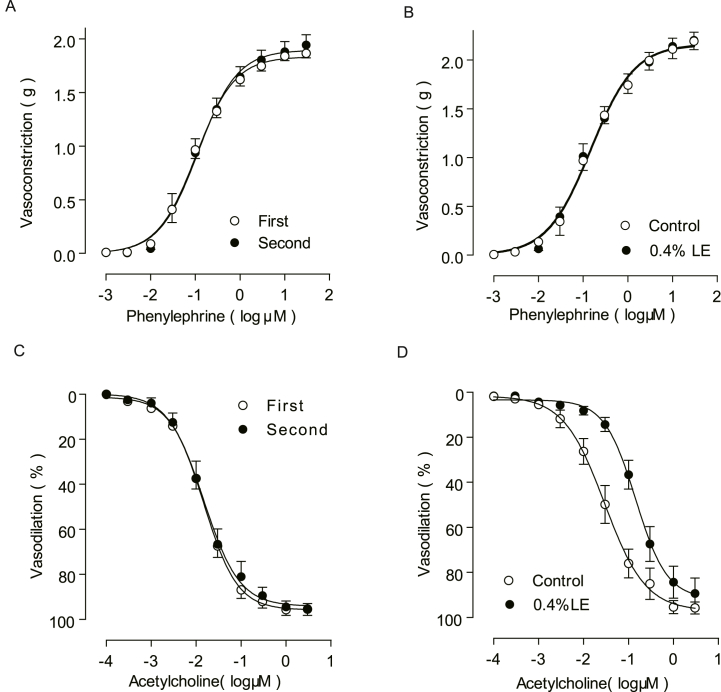
Effect of lipid emulsion (LE) on vascular response in isolated rat aorta ring (A) Repeat vasoconstriction responses to phenylephrine in the isolated rat aorta ring (P > 0.05, n = 8). “First” and “second” indicate the order of the procedure. (B) Vasoconstriction response to phenylephrine in the isolated rat aorta ring following LE pretreatment for 5 min (P > 0.05, n = 8). (C) Repeat vasodilation response to acetylcholine in the isolated rat aorta ring (P > 0.05, n = 8). “First” and “second” indicate the order of the procedure. (D) Vasodilation response to acetylcholine in the isolated rat aorta ring following LE pretreatment for 5 min (*P < 0.05, n = 8). The values are presented as mean ± SEM; n indicates the number of aortic rings from five rats. *P < 0.05 vs. control using two-way ANOVA followed by Bonferroni's post hoc test.

Prior to investigating the impacts of LE on aortic preparations, a repeated Phe dose-response curve range from 0.001 to 30 μmol/L was established to evaluate the functionality of the preparations. A comparison of the two Phe concentration-response curves revealed no significant differences (Fig. 1A). The EC_50_ value for Phe-induced vasoconstriction was determined to be 0.10 ± 0.01 μmol/L. Aortic rings pre-incubated with 0.4 % LE for 5 min exhibited similar levels of Phe-induced vasoconstriction (Fig. 1B) as the control group. The EC_50_ value of Phe-induced vasoconstriction in the presence of LE was 0.12 ± 0.02 μmol/L.

As previously documented, the contracted aortic artery exhibited vasodilation in response to ACh at a concentration range from 0.0001 to 3.0 μmol/L in a dosage-dependent manner. The reproducibility of the ACh dose-response curve is illustrated in Fig. 1C. This procedure was repeated twice to evaluate the function of the preparations. In contrast to Phe-induced vasoconstriction, ACh-mediated vasodilation was significantly impeded by pre-incubation with 0.4 % LE for 5 min (Fig. 1D). The EC_50_ value for Ach-induced vasodilation shifted from 0.03 ± 0.01 μmol/L to 0.14 ± 0.02 μmol/L (P < 0.01).

### 3.2 LE decreased ATP-mediated [Ca^2+^]_i_ elevation in HAECs

In this experiment, incubation with 0.4 % LE influenced ACh-mediated vasodilation. The vasodilatory effect on the aortic artery is contingent upon endothelial cell functionality. Consequently, the intracellular calcium concentration ([Ca^2+^]_i_) in endothelial cells was investigated to elucidate the mechanism of LE-induced vasodilation.

**Fig. 2:**
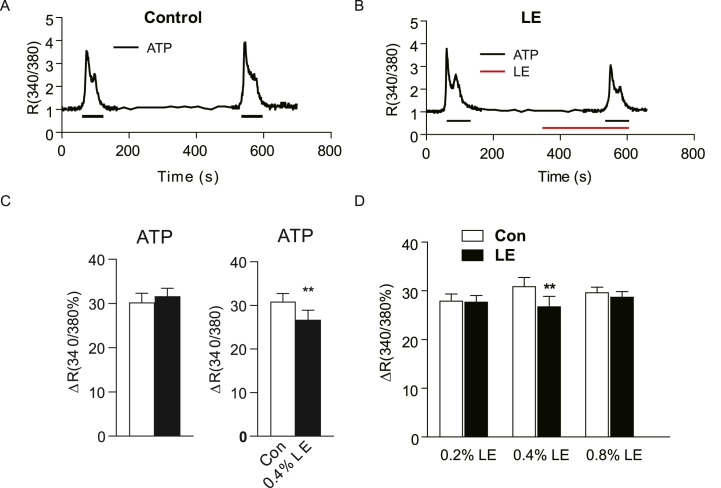
Lipid emulsion (LE, 0.4 %) exerts a suppressive effect on ATP-induced [Ca^2+^]_i_ elevation in human aortic endothelia cells (HAECs). (A) Example calcium imaging recording of HAECs subjected to repeated stimulation with 10 μmol/L ATP for 1 min. (B) Example recording of calcium imaging of HAECs stimulated with 10 μmol/L ATP for 1 min after 0.4 % LE pretreatment for 5 min. (C) Summary graphs of (A) and (B). No significant difference was found after the repeated stimulation with ATP in HAECs (P > 0.05, n = 14). Pretreatment with 0.4 % LE significantly suppressed the calcium upregulation induced by ATP in HAECs (P < 0.01, n = 31). (D) HAECs were stimulated using 10 μmol/L ATP for 1 min after pre-incubation with different concentrations of LE (0.2 %, 0.4 %, 0.8 %) for 5 min (n = 38, 31, 39 for 0.2 %, 0.4 %, 0.8 % LE, respectively). The data are presented as mean ± SEM. Paired *t*-test was employed for the data in C. One-way ANOVA plus Bonferroni's post hoc test was used for the data in D. *P < 0.05, **P < 0.01, ***P < 0.001 vs. control group.

HAECs displayed a spindle-shaped morphology and adhered to the culture flasks under an inverted microscope. Utilizing calcium imaging technology, the [Ca^2+^]_i_ in HAECs was quantified. The results showed that pre-incubation with 0.4 % LE attenuated the increase in [Ca^2+^]_i_ induced by 3 μmol/L ACh in endothelial cells. Owing to a low response to Ach (less than 20 %), the cells were subsequently exposed to ATP, which elicited a more pronounced endothelial cell response. Like ACh, ATP at concentration of 10 μmol/L significantly increased [Ca^2+^]_i_ in HAECs, with the peak increase occurring within 10–15 s of 10 μmol/L ATP stimulation (Fig. 2A). The [Ca^2+^]_i_ peak gradually returned to baseline upon ATP removal (Fig. 2A). The ATP-induced increase in [Ca^2+^]_i_ was consistent and reproducible in the HAECs. Specifically, pre-incubation with 0.4 % LE for 5 min significantly attenuated ATP-induced [Ca^2+^]_i_ elevation in HAECs (Fig. 2B and C).

**Fig. 3:**
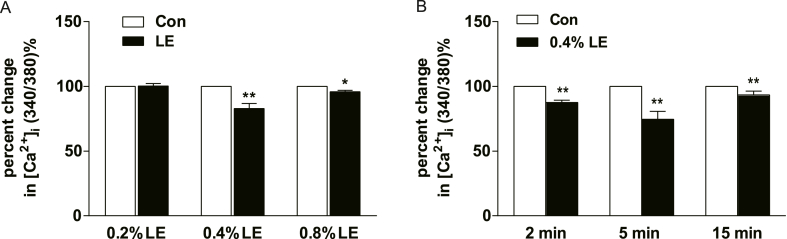
Effect of different concentrations of lipid emulsion (LE) on ATP-induced [Ca^2+^]_i_ increases in HAECs. (A) Normalized data on the effect of pre-incubation with different concentrations of LE (0.2 %, 0.4 %, 0.8 %) for 5 min on intracellular calcium in 10 μmol/L ATP-stimulated HAECs for 1 min. The graph shows no concentration-response relationship (n = 38, 31, 39 for 0.2 %, 0.4 %, 0.8 % LE, respectively). (B) HAECs were incubated with 0.4 % LE for varying durations (2 min, 5 min, 15 min). The data were normalized with control group. The graph shows no time-response relationship (n = 14, 26, 26, for 2 min, 5 min, 15 min, respectively). The data are presented as mean ± SEM. One-way ANOVA plus Bonferroni's post hoc test was used for the data. *P < 0.05, **P < 0.01, vs. control group.

Furthermore, pre-incubation with varying concentrations of LE (0.2 %, 0.4 %, and 0.8 %) for 5 min resulted in differential effects on the ATP-induced [Ca^2+^]_i_ increase in endothelial cells, as depicted in Fig. 2, Fig. 3A. Interestingly, the inhibitory effect of LE pre-incubation on [Ca^2+^]_i_ was not concentration-dependent (Fig. 3A). Notably, the most significant reduction in [Ca^2+^]_i_ was observed after pre-incubation with 0.4 % LE, which is consistent with the recommended maintenance dose of LE for the treatment of local anaesthetic poisoning. Additionally, 0.4 % LE inhibited ATP-induced [Ca^2+^]_i_ elevation at various incubation times (2, 5, and 15 min) in endothelial cells, as displayed in Fig. 3B. However, this inhibition was not time-dependent.

### 3.3 LE decreased ATP-induced [Ca^2+^]_i_ elevation by enhancing calcium uptake into the endoplasmic reticulum in endothelial cells

**Fig. 4:**
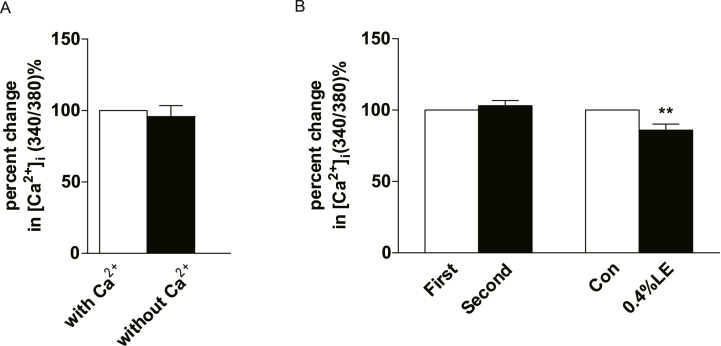
Effect of lipid emulsion (LE) on ATP-induced [Ca^2+^]_i_ increases in HAECs was found to be independent of extracellular Ca^2+^. (A) A comparison of [Ca2+]i responses with and without extracellular Ca^2+^ (EGTA 0.1 mmol L^−1^) in presence of 10 μmol/L ATP reveal no significant difference in HAECs (P > 0.05, n = 26). (B) Repetitive exposure to 10 μmol/L ATP failed to elicit a significant change in [Ca^2+^]_i_ level in the presence of EGTA (P > 0.05, n = 17). “First” and “second” indicate the order of the procedure. Notably, a 5-min pre-incubation with 0.4 % LE for 5 min significantly suppressed the increase of [Ca^2+^]_i_ triggered by 10 μmol/L ATP under the same condition in HAECs (P < 0.01, n = 22). The data are presented as mean ± SEM. Paired *t*-test was employed for the data. **P < 0.01 vs. control group.

To investigate the mechanism by which LE reduces intracellular calcium concentrations, extracellular calcium was removed using the calcium chelator ethylene glycol-bis(2-aminoethylether)-N,N,N′,N′-tetra acetic acid (EGTA; 0.1 mM) in HAECs. Despite its removal, ATP continued to upregulate [Ca^2+^]_i_ in HAECs. Furthermore, there was no significant difference in the ATP-induced [Ca^2+^]_i_ increase after extracellular calcium chelation (Fig. 4A and B), suggesting that ATP-induced calcium elevation primarily relies on intracellular calcium release in HAECs. Additionally, in the presence of EGTA, which chelates extracellular calcium, pre-incubation with 0.4 % LE for 5 min significantly inhibited ATP-mediated [Ca^2+^]_i_ elevation in HAECs (Fig. 4). No significant difference was observed between LE-mediated inhibition with and without the calcium chelator. This indicated that the inhibitory effect of LE on the ATP-induced calcium increase was independent of extracellular calcium influx. The observed inhibition of the ATP-induced calcium increases by LE likely involves a reduction in intracellular calcium release or an enhancement in calcium reuptake into the ER in endothelial cells.

**Fig. 5:**
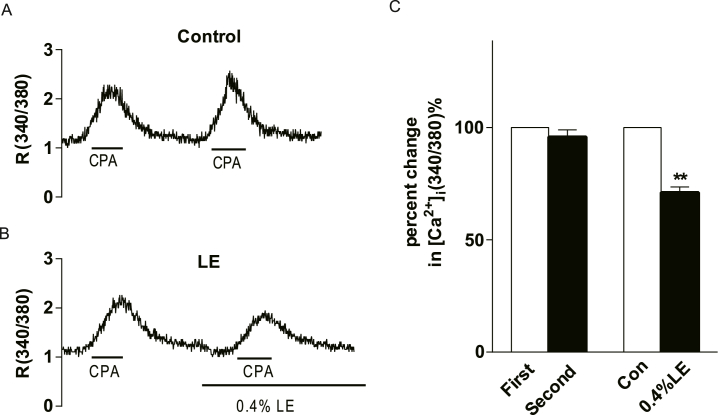
Effect of lipid emulsion (LE) pre-incubation on the cyclopiazonic acid (CPA)-induced calcium increase in HAECs. (A) Example calcium imaging recording of HAECs subjected to repeated stimulation with 5 μmol/L CPA for 1 min. (B) Example recording of calcium imaging of HAECs stimulated with 5 μmol/L CPA for 1 min after 0.4 % LE pretreatment for 5 min. (C) Summary data of A and B. No significant difference was found after the repeated stimulation with CPA in HAECs (P > 0.05, n = 30). Pretreatment with 0.4 % LE significantly suppressed the calcium upregulation induced by CPA in HAECs (P < 0.01, n = 40). The data are presented as mean ± SEM. Paired *t*-test was employed for the data. **P < 0.01 vs. control group.

Given the reliance of intracellular calcium reuptake into the ER on sarcoendoplasmic reticulum Ca^2+^ ATPases (SERCA pumps), an experiment was conducted to investigate the effect of LE on this process. Cyclopiazonic acid (CPA), a SERCA pump inhibitor, was used to evaluate calcium reuptake into the ER. The results showed that CPA at concentration of 5 μmol/L elicited a comparable rise in intracellular free calcium concentrations in endothelial cell as ATP did (Fig. 5A). The analysis revealed no significant difference in calcium elevation following the administration of CPA twice (Fig. 5A–C). Furthermore, pre-incubation with 0.4 % LE for 5 min significantly inhibited the CPA-induced [Ca^2+^]_i_ elevation in endothelial cells (Fig. 5B and C). Consistent with our previous hypothesis, LE may affect [Ca^2+^]_i_ endothelial cells by promoting the reuptake of calcium into the ER.

### 3.4 Effects of LE on nitric oxide (NO) release in cultured vascular endothelial cells

**Fig. 6:**
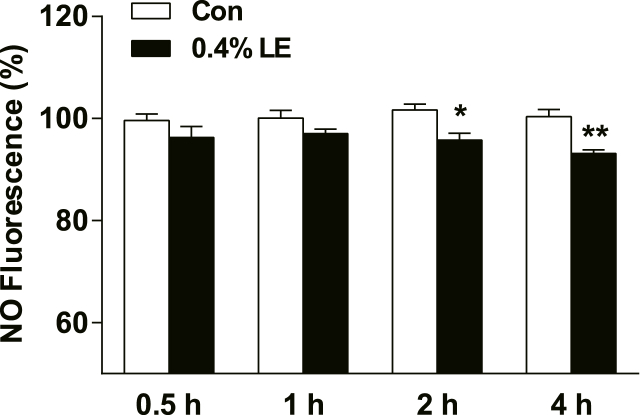
Pre-incubation with 0.4 % LE for varying durations (0.5 h, 1 h, 2 h, 4 h) significantly influenced the nitric oxide (NO) production in response to 10 μmol/L ATP. The data are presented as mean ± SEM. One-way ANOVA plus Bonferroni's post hoc test was used for the data. Paired *t*-test was employed for the same time. *P < 0.05, **P < 0.01 vs control group, n = 5.

In this study, we investigated the impact of LE on NO production using DAF-FM DA (5 μM) as an NO fluorescent probe. Pre-incubation with 0.4 % LE for more than 2 h resulted in a reduction in ATP-induced NO production (Fig. 6). These observations suggest that NO generation was attenuated by decreasing the calcium concentration after incubation with LE.

## 4 Discussion

LE is a common therapeutic approach for managing the systemic toxicity caused by local anaesthetics. Prior studies have demonstrated its ability to alleviate vasodilation associated with local anaesthetics [2]. In this study, LE partially blocked ACh-induced vasodilation, which is consistent with previous findings [12]. Furthermore, LE diminished the rise in cytoplasmic calcium induced by ATP, facilitated the reuptake of calcium into the endoplasmic reticulum, and decreased NO production in HAECs stimulated by ATP. This mechanism may underlie the vasodilation-blocking effects of LE.

LE has been extensively employed as a parenteral nutritional agent in clinics since 1998. Weingberg et al. demonstrated its efficacy in enhancing cardiac resuscitation success rates following local anaesthetic overdoses in rats [2]. Subsequently, LE has been widely adopted to rescue clinical local anaesthetic toxicity, particularly in cases of hypotension, cardiac arrest, and other toxic reactions [4]. The lipid sink theory presumes that LE absorbs anaesthetics and reduces their free blood concentration, which is a prevalent explanation for its therapeutic role [6]. However, recent studies have challenged this notion by revealing that LE counteracts local anaesthetic-induced vasodilation. Moreover, LE significantly enhances the maximum vasoconstriction elicited by norepinephrine [18,19], as observed in clinical applications where it has been shown to elevate blood pressure, an effect that was not observed in this in vitro study [8,[20], [21], [22], [23]]. Guo et al. found that pre-incubation with LE enhanced maximum vasoconstriction (Emax) in response to norepinephrine or Phe, with Emax being time-dependent [12]. Additionally, LE exerted a time-independent inhibitory effect on ACh-induced vasodilation, which was not observed in this study. These findings suggested that the mechanisms underlying the effects of LE on vasoconstriction and vasodilation may differ.

In the present study, a 5-min pre-exposure to 0.4 % LE did not affect Phe-induced vasoconstriction; however, it significantly abolished the vasodilation response to ACh. The ACh-induced vasodilation dose-response curve exhibited a significant shift to the right, indicating that 5 min of LE pre-incubation primarily mitigated vasodilation without affecting vasoconstriction. Given that endothelial integrity is crucial for ACh-induced relaxation in isolated arterial rings [24], these findings suggest that the LE-mediated inhibition of vasorelaxation is endothelium-dependent.

Endothelial cells synthesise and release various vasodilatory substances including NO and prostaglandin I_2_ [13]. NO production is intricately linked to fluctuations in cytoplasmic calcium levels, which are regulated by two primary pathways [25]. First, extracellular calcium enters the cell via calcium-permeable channels such as store-operated cation channels. Normally, extracellular calcium influx into endothelial cells through receptor-operated cation channels is initiated by the release of intracellular Ca^2+^ release [16,17]. However, some channels in the endothelial cell membrane can be directly activated by chemical stimuli, such as the transient receptor potential channel 6 (TRPC6), which is activated by diacylglycerol [16,17]. Second, intracellular calcium is released from calcium stores, such as the ER. Stimulators, such as Ach, ATP, and pressure, can modulate cytoplasmic calcium levels through these two pathways [17,26]. Calcium ions (Ca^2+^) serve as a well-established second messenger in the mediation of calcium-calmodulin (CaM) and protein kinase A activity. An increase in cytoplasmic calcium levels leads to CaM binding and formation of a Ca^2+^/CaM complex. This complex, in turn, activates of endothelial nitric oxide synthase (eNOS) and facilitates NO synthesis [[26], [27],^25^,^28^].

In the present study, calcium alterations in HAECs were assessed using calcium imaging. Our findings revealed that pretreatment with 0.4 % LE significantly attenuated the ACh-induced increase in calcium (Suppl Fig. 1) and ATP levels in endothelial cells. LE at a concentration of 0.4 % demonstrated the most pronounced inhibitory effect, consistent with its known use in treating local anaesthetic toxicity. Given that endothelial cells process both P2X and P2Y purinergic receptors, ATP elicits calcium elevation through a dual mechanism: (1) by activating P2X receptor channels and allowing extracellular calcium influx, and (2) by binding to the P2Y receptor, which releases calcium from the intracellular calcium store into the cytoplasm upon G protein activation. Our results showed no significant differences in the removal of extracellular calcium, indicating that the primary ATP-induced calcium surge lies in the release of cytoplasmic calcium stores, such as those in the ER. LE maintained its inhibitory effect on ATP-induced calcium elevation even in the absence of extracellular calcium, indicating that LE is primarily involved in the regulation of intracellular calcium release or clearance within endothelial cells. Cytoplasmic calcium clearance was notably enhanced by pre-incubation with LE, as indicated by CPA results.

NO induces endothelial cell vasodilation However, factors such as triacylglycerols, fatty acids, and triglycerides can impair NO synthesis [[29], [30], [31], [32]]. Our study found that LE exerted a suppressive effect on the ATP-induced elevation of calcium levels by stimulating ER reuptake of calcium within vascular endothelial cells. Consequently, the reduction in calcium levels results in a decrease in NO production. The hypothesis that pretreatment with LE affects NO generation was supported by the ATP induced NO detection experiment (Fig. 6), which demonstrated a decrease in NO levels upon LE pre-incubation in HAECs, consistent with previous findings [33,34].

## 5 Limitation

This study focused on in vitro experiments, which allowed for a more precise control and observation of specific biological processes. However, the absence of a local anaesthetic toxicity model precludes the direct drawing of a comprehensive conclusion regarding the overall effect and mechanism of LE on blood pressure in these conditions. This is owing to the technical challenges involved in conducting such experiments.

## 6 Conclusion

Our findings revealed that LE inhibits vasodilation by decreasing the ACh- and ATP-stimulated calcium increase, as well as by reducing the production of NO in endothelial cells, which act on smooth muscle to dilate vessels. This mechanism explains LE reversal of the vasodilatory effects of ACh. This would aid in a better understanding of the physiological effects of LE, as well as its potential side effects or complications.

## Ethical statement

This study was reviewed and approved by the Ethics Committee of Hebei Medical University with the approval number: HebMU-20080026 Shijiazhuang, China. Animal care and experimental procedures were performed in accordance with the National Institutes of Health Guide for the Care and Use of Laboratory Animals (2011).

## Data availability statement

All data to support the conclusions have been provided in the manuscript. The original data underlying this article will be shared on reasonable request to the corresponding author.

## CRediT authorship contribution statement

**Ling Chen:** Data curation. **Hui Bai:** Writing – review & editing, Writing – original draft, Conceptualization. **Jing Zhao:** Data curation. **Panpan Zhang:** Investigation, Formal analysis. **Xinhua Zhang:** Writing – review & editing, Methodology. **Dezhi Kong:** Writing – review & editing, Methodology. **Changzheng Dong:** Writing – review & editing, Validation, Formal analysis. **Wei Zhang:** Writing – review & editing, Supervision, Funding acquisition, Formal analysis, Conceptualization.

## Declaration of competing interest

The authors declare that there are no competing financial interests or personal relationships that could have appeared to influence the work reported in this paper.
