# Supplementary material for: Lipid emulsion attenuates vasodilation by decreasing intracellular calcium and nitric oxide in vascular endothelial cells
Source: Heliyon. 2024 Sep 3;10(17):e37353. doi: 10.1016/j.heliyon.2024.e37353 (PMC11408769; doi:10.1016/j.heliyon.2024.e37353)
Supplement: Multimedia component 1 [file mmc1.docx]

**Supplementary Figure 1.** 0.4% lipid emulsion (LE) exerts a suppressive effect on Ach-evoked [Ca^2+^]_i_ elevation in human aortic endothelia cells (HAECs). (A) Example calcium imaging recording of HAECs were subjected to repeated stimulation with 3 μmol/L Ach for 1 minute. (B) Example recording of calcium imaging of HAECs were stimulated with 3 μmol/L Ach for 1 minute after 0.4% LE pretreatment for 5 minutes. (C) and (D) Summary graphs of (A) and (B). (C) No significant difference was found after the repeated stimulation with Ach in HAECs (P>0.05, n=4). First and second indicated the repeat stimulation with Ach in HAECs. (D) Pretreatment with 0.4% LE significantly suppressed the calcium upregulation induced by Ach in HAECs (P<0.05, n=9). The data are presented as mean ± SEM. Paired t-test was employed for the data in C and D. **P* < 0.05 vs. control group.


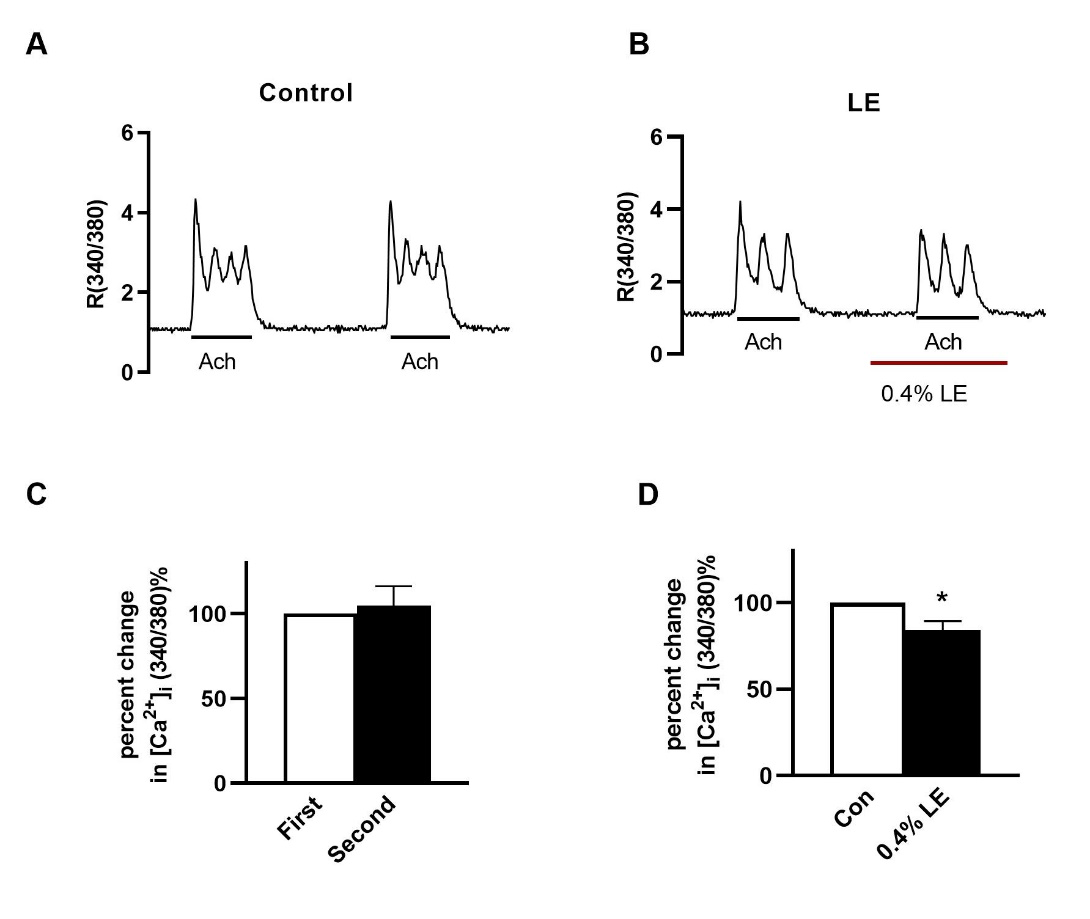


Supplementary figure 1
